# Bis(1-benzoyl-7-meth­oxy­naphthalen-2-yl) terephthalate

**DOI:** 10.1107/S1600536813007186

**Published:** 2013-03-20

**Authors:** Rei Sakamoto, Daichi Hijikata, Katsuhiro Isozaki, Noriyuki Yonezawa, Akiko Okamoto

**Affiliations:** a Department of Organic and Polymer Materials Chemistry, Tokyo University of Agriculture & Technology, Naka-machi, Koganei, Tokyo 184-8588, Japan; b International Research Center for Elements Science, Institute for Chemical Research, Kyoto University, Gokasho, Uji, Kyoto 611-0011, Japan

## Abstract

The title molecule, C_44_H_30_O_8_, lies about a crystallographic inversion centre located at the centre of the central benzene ring. The benzene rings in the benzoyl and the terephthalate units make dihedral angles of 67.05 (7)° and 57.57 (7)°, respectively, with the naphthalene ring system. There is an intra­molecular C—H⋯O inter­action between the ketonic carbonyl O atom and an H atom on the naphthalene ring system. In the crystal, C—H⋯O inter­action of the benzene ring in the benzoyl group and weak C=O⋯π inter­action [O⋯centroid = 3.375 (2) Å] of the naphthalene ring with the O atom in the ketonic carbonyl group are observed. These inter­actions form layers parallel to the *bc* plane.

## Related literature

For electrophilic aromatic aroylation of the naphthalene core, see: Okamoto & Yonezawa (2009 ▶); Okamoto *et al.* (2011 ▶). For the structures of closely related compounds, see: Kato *et al.* (2010 ▶); Nakaema *et al.* (2008 ▶); Sakamoto *et al.* (2012 ▶, 2013 ▶).
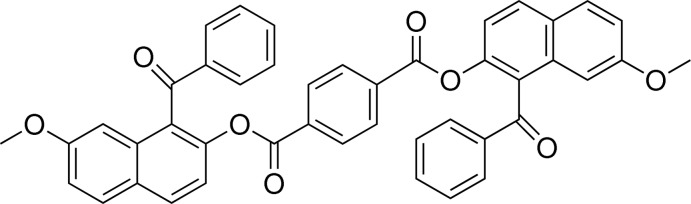


## Experimental

### 

#### Crystal data

C_44_H_30_O_8_
*M* *_r_* = 686.72
Monoclinic, 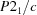

*a* = 9.977 (5) Å
*b* = 14.922 (7) Å
*c* = 11.709 (6) Å
β = 106.610 (5)°
*V* = 1670.5 (14) Å^3^
*Z* = 2
Mo *K*α radiation
μ = 0.09 mm^−1^
*T* = 173 K
0.16 × 0.13 × 0.03 mm

#### Data collection

Rigaku Saturn70 diffractometer
Absorption correction: numerical (*NUMABS*; Higashi, 1999 ▶) *T*_min_ = 0.985, *T*_max_ = 0.997
10969 measured reflections
2909 independent reflections
2354 reflections with *I* > 2σ(*I*)
*R*_int_ = 0.048

#### Refinement

*R*[*F* ^2^ > 2σ(*F* ^2^)] = 0.044
*wR*(*F* ^2^) = 0.122
*S* = 1.04
2909 reflections
236 parameters
H-atom parameters constrained
Δρ_max_ = 0.18 e Å^−3^
Δρ_min_ = −0.18 e Å^−3^

### 

Data collection: *CrystalClear* (Rigaku, 2006 ▶); cell refinement: *CrystalClear*; data reduction: *CrystalClear*; program(s) used to solve structure: *SHELXS97* (Sheldrick, 2008 ▶); program(s) used to refine structure: *SHELXL97* (Sheldrick, 2008 ▶); molecular graphics: *CrystalStructure* (Rigaku, 2010 ▶); software used to prepare material for publication: *CrystalStructure*.

## Supplementary Material

Crystal structure: contains datablock(s) I, New_Global_Publ_Block. DOI: 10.1107/S1600536813007186/rn2113sup1.cif

Structure factors: contains datablock(s) I. DOI: 10.1107/S1600536813007186/rn2113Isup2.hkl

Supplementary material file. DOI: 10.1107/S1600536813007186/rn2113Isup3.cml

Additional supplementary materials:  crystallographic information; 3D view; checkCIF report

## Figures and Tables

**Table 1:** Hydrogen-bond geometry (Å, °)

*D*—H⋯*A*	*D*—H	H⋯*A*	*D*⋯*A*	*D*—H⋯*A*
C8—H8⋯O1	0.95	2.41	2.965 (3)	117
C16—H16⋯O1^i^	0.95	2.55	3.258 (3)	132

Symmetry code: (i) 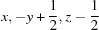.

**Fig. 1.**
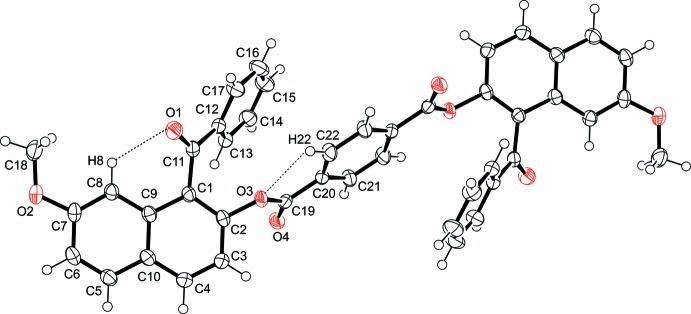
Molecular structure with displacement ellipsoids at 50% probability for non-H atoms. The dashed lines indicate intramolecular C–H···O bonds.

**Fig. 2.**
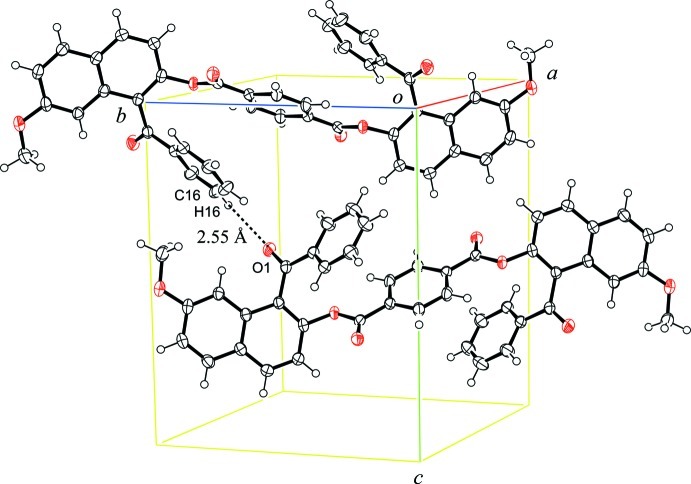
C–H···O interaction (dashed line) between phenyl ring and ketonic carbonyl group.

**Fig. 3.**
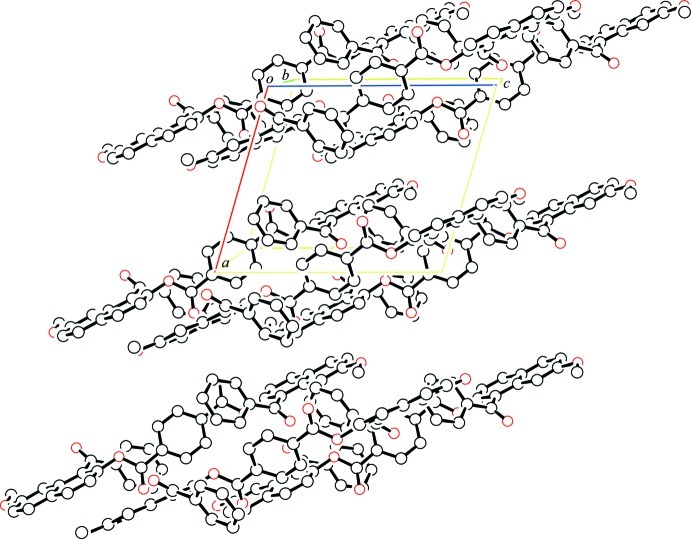
The crystal packing of the title compound, viewed along the b axis. The molecular layers are expanded along the bc-plane.

## supplementary crystallographic information

### Comment

In the course of our study on electrophilic aromatic aroylation of the naphthalene core, 1,8-diaroylnaphthalene compounds have proved to be formed regioselectively by the aid of a suitable acidic mediator (Okamoto & Yonezawa, 2009, Okamoto *et al.*, 2011).

Recently, we have reported the X-ray crystal structures of 1,8-diaroylated 2,7-dialkoxynaphthalene, *e.g.*, 1,8-dibenzoyl-2,7-dimethoxynaphthalene (Nakaema *et al.*, 2008). Furthermore, we have also determined the crystal structures of 1-monoaroylated 2,7-dialkoxynaphthalene compounds such as (2,7-dimethoxynaphthalen-1-yl)(phenyl)methanone [1-benzoyl-2,7-dimethoxynaphthalene](Kato *et al.*, 2010).

These compounds have non-coplanar structures where the aroyl groups are perpendicularly orientated relative to the naphthalene ring. Crystal structures of the aroylnaphthalene analogues bearing oxybenzoyl groups at the 2,7- positions of the naphthalene ring core namely 1,8-dibenzoylnaphthalene-2,7-diyl dibenzoate (Sakamoto *et al.*, 2012) and 1-benzoylnaphthalene-2,7-diyl dibenzoate (Sakamoto *et al.*, 2013), have been previously determined which show the molecules form the tubular arrangements when the benzene ring of the benzoyl group effectively interacts with the carbonyl moiety of the benzoyloxy group and the naphthalene ring through intermolecular C–H···O and C–H···π interactions.

As a part of our ongoing studies on the molecular structures of these kinds of homologous molecules, the X-ray crystal structure of the title compound composed of two 1-benzoylnaphthalene units and a terephthalate moiety is reported on herein.

The molecular structure of the title compound is displayed in Fig. 1. The molecule lies on a centre of inversion so that the asymmetric unit contains one-half of the molecules. The benzene rings in the benzoyl group and the terephthalate moiety are twisted away from the naphthalene ring. Two kinds of intramolecular C–H···O interactions, one intramolecular C–H···O interaction between the naphthalene ring and the benzoyl group (C8–H8···O1 = 2.41 Å) and another one between the benzene ring and the ethereal oxygen of the terephthalate moiety (C22–H22···O3 = 2.39 Å), contribute to stabilization of the twisted orientation of each benzene ring against the naphthalene ring (Fig. 1).

The dihedral angles of the benzene ring in the benzoyl group and the terephthalate moiety with the naphthalene ring are 67.05 (7)° [C9–C1–C11–O1 and O1–C11–C12–C17, torsion angles = -45.1 (3) and -26.3 (3)° for benzoyl group] and 57.57 (7)° [O4–C19–C20–C21, O4–C19–O3–C2, and C3–C2–O3–C19, torsion angles = 2.9 (3), 4.0 (5), and -66.8 (9)° for terephthalate moiety].

In the case of the homologous molecule, 1-benzoylnaphthalene-2,7-diyl dibenzoate (Sakamoto *et al.*, 2013), the corresponding dihedral angles are slightly larger than those of the title compound [80.41 (6)° and 73.62 (5)°].

In the crystal (Fig. 2), the ketonic carbonyl oxygen forms intermolecular C–H···O interaction with the benzene ring of the benzoyl goup [C16–H16···O1^i^ = 2.55 Å; symmetry code: *x*, 1/2 - *y*, -1/2 + *z*] and weak intermolecular C=O···π interaction with the naphthalene ring [C11–O1···*Cg*1^ii^ = 3.38 Å; *Cg*1 is the centroids of the C1/C4–C9–C10 rings].

Consequently, the molecules are arranged in laminae along the *bc*-plane (Fig. 3).

### Experimental

The title compound was prepared by treatment of a mixture of (2-hydroxy-7-methoxynaphthalen-1-yl)(phenyl)methanone (0.4 mmol, 111 mg), terephthaloyl dichloride (0.22 mmol, 44.7 mg), and triethylamine (0.44 mmol, 0.062 ml) in dichloromethane (1.0 ml). After the reaction mixture was stirred at rt for 3 h, it was poured into water (30 ml) and the mixture was extracted with chloroform (10 ml×3). The combined extracts were washed with brine. The organic layers thus obtained were dried over anhydrous MgSO_4_. The solvent was removed under reduced pressure to give cake (yield 63%). The crude product was purified by recrystallization from chloroform and colorless single crystals suitable for X-ray diffraction were obtained.

Spectroscopic data: ^1^H NMR δ (500 MHz, CDCl_3_): 3.76 (6*H*, s), 7.02 (2*H*, dd, *J*=9.2, 2.3 Hz), 7.20 (2*H*, dd, *J*=9.2, 2.3 Hz), 7.33–7.39 (6*H*, m), 7.50 (2*H*, t, *J*=7.4 Hz), 7.66 (4*H*, s), 7.81–7.85 (6*H*, m), 7.96 (2*H*, d, *J*=8.6 Hz) p.p.m..

^13^C NMR δ (75 MHz, CDCl_3_): 55.30, 103.37, 118.54, 119.32, 126.63, 127.18, 128.68, 129.43, 129.71, 129.83, 130.95, 132.84, 133.75, 137.78, 146.33, 158.99, 163.38, 195.98 p.p.m..

IR (KBr): 1729 (OC=O), 1663 (C=O), 1624,1595, 1510 (Ar) cm^-1^. m.p. = 484.2–484.8 K.

Anal. Calcd for C_44_H_30_O_8_ 3.5H_2_O: C, 70.49; H, 4.97; Found: C, 70.40; H, 4.73.

### Refinement

All H atoms were found in a difference map and were subsequently refined as riding atoms, with C—H = 0.95 (aromatic) and 0.98 (methyl) Å with *U*_iso_(H) = 1.2*U*_eq_(C).

### Crystal data

**Table:**

C_44_H_30_O_8_	*F*(000) = 716
*M**_r_* = 686.72	*D*_x_ = 1.365 Mg m^−^^3^
Monoclinic, *P*2_1_/*c*	Mo *K*α radiation, λ = 0.71070 Å
Hall symbol: -P 2ybc	Cell parameters from 4873 reflections
*a* = 9.977 (5) Å	θ = 2.1–31.2°
*b* = 14.922 (7) Å	µ = 0.09 mm^−^^1^
*c* = 11.709 (6) Å	*T* = 173 K
β = 106.610 (5)°	Block, colorless
*V* = 1670.5 (14) Å^3^	0.16 × 0.13 × 0.03 mm
*Z* = 2	

### Data collection

**Table:**

Rigaku Saturn70 diffractometer	2909 independent reflections
Radiation source: fine-focus sealed tube	2354 reflections with *I* > 2σ(*I*)
Graphite monochromator	*R*_int_ = 0.048
Detector resolution: 7.314 pixels mm^-1^	θ_max_ = 25.0°, θ_min_ = 3.3°
ω scans	*h* = −11→11
Absorption correction: numerical (*NUMABS*; Higashi, 1999)	*k* = −13→17
*T*_min_ = 0.985, *T*_max_ = 0.997	*l* = −13→13
10969 measured reflections	

### Refinement

**Table:**

Refinement on *F*^2^	Primary atom site location: structure-invariant direct methods
Least-squares matrix: full	Secondary atom site location: difference Fourier map
*R*[*F*^2^ > 2σ(*F*^2^)] = 0.044	Hydrogen site location: inferred from neighbouring sites
*wR*(*F*^2^) = 0.122	H-atom parameters constrained
*S* = 1.04	*w* = 1/[σ^2^(*F*_o_^2^) + (0.0716*P*)^2^ + 0.0599*P*] where *P* = (*F*_o_^2^ + 2*F*_c_^2^)/3
2909 reflections	(Δ/σ)_max_ < 0.001
236 parameters	Δρ_max_ = 0.18 e Å^−^^3^
0 restraints	Δρ_min_ = −0.18 e Å^−^^3^

### Special details

**Table:**

Geometry. All e.s.d.'s (except the e.s.d. in the dihedral angle between two l.s. planes) are estimated using the full covariance matrix. The cell e.s.d.'s are taken into account individually in the estimation of e.s.d.'s in distances, angles and torsion angles; correlations between e.s.d.'s in cell parameters are only used when they are defined by crystal symmetry. An approximate (isotropic) treatment of cell e.s.d.'s is used for estimating e.s.d.'s involving l.s. planes.
Refinement. Refinement of *F*^2^ against ALL reflections. The weighted *R*-factor *wR* and goodness of fit *S* are based on *F*^2^, conventional *R*-factors *R* are based on *F*, with *F* set to zero for negative *F*^2^. The threshold expression of *F*^2^ > σ(*F*^2^) is used only for calculating *R*-factors(gt) *etc*. and is not relevant to the choice of reflections for refinement. *R*-factors based on *F*^2^ are statistically about twice as large as those based on *F*, and *R*- factors based on ALL data will be even larger.

### Fractional atomic coordinates and isotropic or equivalent isotropic displacement parameters (Å^2^)

**Table:**

	*x*	*y*	*z*	*U*_iso_*/*U*_eq_	
O1	0.89735 (14)	0.10507 (8)	0.99483 (11)	0.0365 (3)	
O2	0.58268 (14)	0.02769 (9)	1.24046 (11)	0.0370 (4)	
O3	0.88903 (12)	−0.06534 (8)	0.75583 (10)	0.0274 (3)	
O4	0.71518 (13)	−0.10741 (8)	0.59275 (11)	0.0331 (3)	
C1	0.78482 (18)	−0.02789 (11)	0.90842 (14)	0.0250 (4)	
C2	0.81191 (18)	−0.09200 (11)	0.83329 (15)	0.0268 (4)	
C3	0.78049 (19)	−0.18316 (12)	0.83991 (16)	0.0300 (4)	
H3	0.8021	−0.2253	0.7870	0.036*	
C4	0.71849 (19)	−0.20981 (12)	0.92361 (16)	0.0317 (4)	
H4	0.6966	−0.2714	0.9288	0.038*	
C5	0.6193 (2)	−0.17586 (12)	1.08887 (16)	0.0323 (4)	
H5	0.5964	−0.2373	1.0932	0.039*	
C6	0.58792 (19)	−0.11631 (12)	1.16488 (16)	0.0328 (4)	
H6	0.5437	−0.1363	1.2221	0.039*	
C7	0.62077 (19)	−0.02485 (12)	1.15912 (15)	0.0298 (4)	
C8	0.68635 (18)	0.00528 (12)	1.07862 (14)	0.0268 (4)	
H8	0.7093	0.0670	1.0768	0.032*	
C9	0.72028 (18)	−0.05563 (11)	0.99763 (15)	0.0263 (4)	
C10	0.68584 (18)	−0.14785 (11)	1.00309 (15)	0.0286 (4)	
C11	0.83238 (18)	0.06688 (11)	0.90305 (15)	0.0273 (4)	
C12	0.79951 (18)	0.11478 (11)	0.78599 (15)	0.0272 (4)	
C13	0.68449 (19)	0.09267 (11)	0.69170 (16)	0.0295 (4)	
H13	0.6248	0.0451	0.7002	0.035*	
C14	0.6557 (2)	0.13957 (12)	0.58465 (17)	0.0366 (5)	
H14	0.5757	0.1250	0.5208	0.044*	
C15	0.7446 (2)	0.20763 (13)	0.57206 (18)	0.0426 (5)	
H15	0.7262	0.2393	0.4988	0.051*	
C16	0.8603 (2)	0.22994 (13)	0.66549 (19)	0.0425 (5)	
H16	0.9210	0.2765	0.6558	0.051*	
C17	0.8877 (2)	0.18470 (12)	0.77284 (18)	0.0365 (5)	
H17	0.9660	0.2010	0.8374	0.044*	
C18	0.6184 (2)	0.12025 (13)	1.24527 (18)	0.0409 (5)	
H18A	0.5809	0.1469	1.1661	0.049*	
H18B	0.7204	0.1267	1.2711	0.049*	
H18C	0.5784	0.1509	1.3020	0.049*	
C19	0.82851 (18)	−0.07386 (11)	0.63596 (15)	0.0259 (4)	
C20	0.91934 (18)	−0.03603 (11)	0.56745 (15)	0.0256 (4)	
C21	0.87490 (19)	−0.04257 (12)	0.44403 (16)	0.0306 (4)	
H21	0.7890	−0.0716	0.4059	0.037*	
C22	1.04521 (19)	0.00720 (12)	0.62350 (15)	0.0316 (4)	
H22	1.0756	0.0122	0.7079	0.038*	

### Atomic displacement parameters (Å^2^)

**Table:**

	*U*^11^	*U*^22^	*U*^33^	*U*^12^	*U*^13^	*U*^23^
O1	0.0435 (8)	0.0394 (8)	0.0269 (7)	−0.0090 (6)	0.0105 (6)	−0.0069 (6)
O2	0.0448 (8)	0.0425 (8)	0.0294 (7)	0.0008 (6)	0.0198 (6)	−0.0037 (5)
O3	0.0321 (7)	0.0316 (7)	0.0211 (6)	−0.0011 (5)	0.0120 (5)	−0.0004 (5)
O4	0.0366 (8)	0.0359 (7)	0.0287 (7)	−0.0071 (6)	0.0124 (6)	−0.0030 (5)
C1	0.0281 (9)	0.0274 (9)	0.0202 (9)	0.0003 (7)	0.0080 (7)	0.0017 (7)
C2	0.0286 (9)	0.0326 (10)	0.0206 (9)	0.0002 (7)	0.0092 (7)	0.0035 (7)
C3	0.0372 (10)	0.0271 (10)	0.0281 (10)	0.0013 (7)	0.0130 (8)	−0.0007 (7)
C4	0.0399 (11)	0.0252 (9)	0.0319 (10)	0.0005 (8)	0.0133 (8)	0.0024 (7)
C5	0.0364 (10)	0.0310 (10)	0.0311 (10)	0.0000 (8)	0.0122 (8)	0.0063 (8)
C6	0.0346 (10)	0.0403 (11)	0.0267 (10)	0.0005 (8)	0.0140 (8)	0.0073 (8)
C7	0.0298 (10)	0.0399 (11)	0.0211 (9)	0.0052 (8)	0.0097 (7)	0.0006 (7)
C8	0.0291 (9)	0.0303 (9)	0.0213 (9)	0.0016 (7)	0.0074 (7)	0.0012 (7)
C9	0.0268 (9)	0.0304 (10)	0.0211 (9)	0.0016 (7)	0.0059 (7)	0.0036 (7)
C10	0.0307 (10)	0.0301 (10)	0.0253 (9)	0.0000 (7)	0.0086 (8)	0.0046 (7)
C11	0.0283 (9)	0.0301 (10)	0.0262 (9)	−0.0003 (7)	0.0125 (8)	−0.0014 (7)
C12	0.0316 (10)	0.0254 (9)	0.0275 (10)	0.0023 (7)	0.0131 (8)	0.0004 (7)
C13	0.0327 (10)	0.0269 (9)	0.0314 (10)	0.0016 (7)	0.0134 (8)	0.0002 (7)
C14	0.0411 (11)	0.0342 (11)	0.0329 (11)	0.0030 (9)	0.0079 (9)	0.0040 (8)
C15	0.0561 (13)	0.0356 (11)	0.0366 (12)	0.0030 (10)	0.0143 (10)	0.0131 (8)
C16	0.0475 (12)	0.0329 (11)	0.0503 (13)	−0.0046 (9)	0.0191 (10)	0.0111 (9)
C17	0.0382 (11)	0.0331 (10)	0.0395 (11)	−0.0045 (8)	0.0131 (9)	0.0029 (8)
C18	0.0505 (13)	0.0428 (12)	0.0333 (11)	0.0048 (9)	0.0182 (10)	−0.0040 (8)
C19	0.0325 (10)	0.0227 (9)	0.0240 (9)	0.0024 (7)	0.0105 (8)	−0.0018 (7)
C20	0.0300 (9)	0.0240 (9)	0.0253 (9)	0.0027 (7)	0.0121 (7)	0.0007 (7)
C21	0.0307 (10)	0.0370 (10)	0.0261 (10)	−0.0051 (8)	0.0110 (8)	−0.0023 (7)
C22	0.0366 (10)	0.0392 (10)	0.0190 (9)	−0.0029 (8)	0.0078 (8)	−0.0016 (8)

### Geometric parameters (Å, º)

**Table:**

O1—C11	1.225 (2)	C11—C12	1.497 (2)
O2—C7	1.369 (2)	C12—C13	1.386 (3)
O2—C18	1.423 (2)	C12—C17	1.401 (3)
O3—C19	1.366 (2)	C13—C14	1.392 (3)
O3—C2	1.404 (2)	C13—H13	0.9500
O4—C19	1.208 (2)	C14—C15	1.384 (3)
C1—C2	1.378 (2)	C14—H14	0.9500
C1—C9	1.436 (2)	C15—C16	1.385 (3)
C1—C11	1.499 (2)	C15—H15	0.9500
C2—C3	1.403 (3)	C16—C17	1.383 (3)
C3—C4	1.359 (2)	C16—H16	0.9500
C3—H3	0.9500	C17—H17	0.9500
C4—C10	1.414 (3)	C18—H18A	0.9800
C4—H4	0.9500	C18—H18B	0.9800
C5—C6	1.356 (3)	C18—H18C	0.9800
C5—C10	1.416 (2)	C19—C20	1.483 (2)
C5—H5	0.9500	C20—C21	1.389 (3)
C6—C7	1.410 (3)	C20—C22	1.397 (3)
C6—H6	0.9500	C21—C22^i^	1.378 (3)
C7—C8	1.368 (2)	C21—H21	0.9500
C8—C9	1.422 (2)	C22—C21^i^	1.378 (3)
C8—H8	0.9500	C22—H22	0.9500
C9—C10	1.424 (3)		
			
C7—O2—C18	117.86 (14)	C13—C12—C11	122.05 (16)
C19—O3—C2	118.32 (13)	C17—C12—C11	118.43 (16)
C2—C1—C9	118.35 (15)	C12—C13—C14	120.55 (17)
C2—C1—C11	120.41 (15)	C12—C13—H13	119.7
C9—C1—C11	121.05 (15)	C14—C13—H13	119.7
C1—C2—C3	123.39 (16)	C15—C14—C13	119.44 (18)
C1—C2—O3	117.35 (15)	C15—C14—H14	120.3
C3—C2—O3	118.86 (15)	C13—C14—H14	120.3
C4—C3—C2	118.58 (16)	C14—C15—C16	120.47 (18)
C4—C3—H3	120.7	C14—C15—H15	119.8
C2—C3—H3	120.7	C16—C15—H15	119.8
C3—C4—C10	121.40 (17)	C17—C16—C15	120.28 (19)
C3—C4—H4	119.3	C17—C16—H16	119.9
C10—C4—H4	119.3	C15—C16—H16	119.9
C6—C5—C10	121.01 (17)	C16—C17—C12	119.72 (19)
C6—C5—H5	119.5	C16—C17—H17	120.1
C10—C5—H5	119.5	C12—C17—H17	120.1
C5—C6—C7	120.15 (16)	O2—C18—H18A	109.5
C5—C6—H6	119.9	O2—C18—H18B	109.5
C7—C6—H6	119.9	H18A—C18—H18B	109.5
O2—C7—C8	125.02 (17)	O2—C18—H18C	109.5
O2—C7—C6	114.00 (15)	H18A—C18—H18C	109.5
C8—C7—C6	120.98 (16)	H18B—C18—H18C	109.5
C7—C8—C9	120.07 (17)	O4—C19—O3	123.71 (16)
C7—C8—H8	120.0	O4—C19—C20	125.10 (16)
C9—C8—H8	120.0	O3—C19—C20	111.19 (14)
C8—C9—C10	118.76 (15)	C21—C20—C22	119.86 (16)
C8—C9—C1	122.84 (16)	C21—C20—C19	118.26 (16)
C10—C9—C1	118.38 (15)	C22—C20—C19	121.86 (16)
C4—C10—C5	121.11 (16)	C22^i^—C21—C20	120.34 (17)
C4—C10—C9	119.88 (16)	C22^i^—C21—H21	119.8
C5—C10—C9	119.02 (16)	C20—C21—H21	119.8
O1—C11—C12	120.23 (16)	C21^i^—C22—C20	119.80 (17)
O1—C11—C1	119.77 (16)	C21^i^—C22—H22	120.1
C12—C11—C1	120.00 (15)	C20—C22—H22	120.1
C13—C12—C17	119.52 (17)		
			
C9—C1—C2—C3	−0.4 (3)	C8—C9—C10—C5	−0.1 (2)
C11—C1—C2—C3	174.71 (16)	C1—C9—C10—C5	−178.62 (16)
C9—C1—C2—O3	−173.00 (14)	C2—C1—C11—O1	−129.92 (19)
C11—C1—C2—O3	2.1 (2)	C9—C1—C11—O1	45.1 (2)
C19—O3—C2—C1	−120.17 (17)	C2—C1—C11—C12	50.6 (2)
C19—O3—C2—C3	66.9 (2)	C9—C1—C11—C12	−134.45 (17)
C1—C2—C3—C4	0.9 (3)	O1—C11—C12—C13	−152.96 (17)
O3—C2—C3—C4	173.35 (16)	C1—C11—C12—C13	26.6 (2)
C2—C3—C4—C10	−0.1 (3)	O1—C11—C12—C17	26.3 (2)
C10—C5—C6—C7	0.3 (3)	C1—C11—C12—C17	−154.14 (16)
C18—O2—C7—C8	−2.5 (3)	C17—C12—C13—C14	−0.3 (3)
C18—O2—C7—C6	176.79 (16)	C11—C12—C13—C14	178.96 (16)
C5—C6—C7—O2	179.69 (17)	C12—C13—C14—C15	1.2 (3)
C5—C6—C7—C8	−1.0 (3)	C13—C14—C15—C16	−0.9 (3)
O2—C7—C8—C9	−179.64 (16)	C14—C15—C16—C17	−0.4 (3)
C6—C7—C8—C9	1.1 (3)	C15—C16—C17—C12	1.3 (3)
C7—C8—C9—C10	−0.6 (2)	C13—C12—C17—C16	−1.0 (3)
C7—C8—C9—C1	177.87 (16)	C11—C12—C17—C16	179.72 (17)
C2—C1—C9—C8	−179.22 (16)	C2—O3—C19—O4	−4.1 (2)
C11—C1—C9—C8	5.7 (3)	C2—O3—C19—C20	175.43 (13)
C2—C1—C9—C10	−0.8 (2)	O4—C19—C20—C21	−2.9 (3)
C11—C1—C9—C10	−175.87 (15)	O3—C19—C20—C21	177.60 (14)
C3—C4—C10—C5	179.04 (17)	O4—C19—C20—C22	175.60 (17)
C3—C4—C10—C9	−1.1 (3)	O3—C19—C20—C22	−3.9 (2)
C6—C5—C10—C4	−179.90 (17)	C22—C20—C21—C22^i^	0.5 (3)
C6—C5—C10—C9	0.2 (3)	C19—C20—C21—C22^i^	179.03 (16)
C8—C9—C10—C4	−179.97 (16)	C21—C20—C22—C21^i^	−0.5 (3)
C1—C9—C10—C4	1.5 (2)	C19—C20—C22—C21^i^	−178.97 (16)

Symmetry code: (i) −*x*+2, −*y*, −*z*+1.

### Hydrogen-bond geometry (Å, º)

**Table:**

*D*—H···*A*	*D*—H	H···*A*	*D*···*A*	*D*—H···*A*
C8—H8···O1	0.95	2.41	2.965 (3)	117
C16—H16···O1^ii^	0.95	2.55	3.258 (3)	132
C22—H22···O3	0.95	2.39	2.717 (3)	100

Symmetry code: (ii) *x*, −*y*+1/2, *z*−1/2.
